# Local drug delivery agents as adjuncts to endodontic and periodontal therapy

**Published:** 2013-12-25

**Authors:** K Puri, N Puri

**Affiliations:** * Department of Periodontics, Institute of Dental Studies and Technologies; ** Department of Endodontics, Institute of Dental Studies and Technologies Kadrabad, Modinagar, Ghaziabad

**Keywords:** Periodontal pocket, periodontal therapy, root canal, endodontic failures, local delivery systems

## Abstract

Abstract

In the treatment of intracanal and periodontal infections, the local application of antibiotics and other therapeutic agents in the root canal or in periodontal pockets may be a promising approach to achieve sustained/controlled drug release, high antimicrobial activity and low systemic side effects. The conventional method for the elimination of subgingival microbial infection includes mechanical debridement, irrigation with antimicrobial agents or surgical access. But, the effectiveness of conventional nonsurgical treatment is limited by lack of accessibility to bacteria in deeper periodontal pockets, and/or does not completely eliminate intracanal microorganisms. Surgical intervention may be beneficial but cannot be done in all cases, medically compromised cases and also in patients not willing to be subjected to surgical therapy. Development of local drug delivery systems provides an answer to all such difficulties. This comprehensive review tries to cover the detailed information about the latest advances in the various local drug delivery systems, their indications, contraindications and their advantages over systemic drug therapy.

## Introduction

Periodontal and endodontic pathologies are closely related to each other and are the most common dental diseases leading to tooth loss. Periodontal diseases are bacterial infections characterized by inflammation and destruction of the attachment apparatus, often leading to tooth loss. The most common forms of periodontal diseases, gingivitis and periodontitis are caused by bacteria adjacent to or associated with periodontal structures. These bacteria, along with calculus and other local factors, are the principal components that perpetuate the disease process [**1**].

Plaque constitutes highly organized bacterial populations. Elevated proportions of some subgingival microbial species have been associated with destructive periodontal disease activity. Potential periodontal pathogens include Aggregtibacter actinomycetemcomitans, Porphyromonas gingivalis, Prevotella intermedia, Tannerella forsythus, Peptostreptococcous micros, Campylobacter rectus, Eiknella corrodens, Fusobacterium nucleatum, Eubacterium species, Treponema denticola, Beta-hemolytic streptococci, a variety of enteric rods and pseudomonas, Enterococci, Staphylococci and possibly yeasts [**2**,**3**]. It is important to keep the pathogenic microflora of the pocket suppressed in order to maintain health of the periodontal tissues [**4**,**5**]. Modalities of treatment may be a non surgical approach or a surgical approach [**4**].

Debridement of the root surface by scaling and root planing came into a relatively common use in the first half of the past century and has become the central feature held in common by all currently used forms of periodontal therapy [**6**].

Scaling and root planing has been effective in immediately decreasing the microbial load but recolonization of the same can occur as early as 60 days after scaling and root planing. Hence, chemical therapy has been advocated to inhibit pathogenic microflora and encourage inhibition of non pathogenic microflora [**4**,**7**-**9**]. Endodontic biofilms represent a common cause of persistent infections in the root canal because of their resistance to antimicrobial compounds [**10**]. It is of particular concern that resistant microbial species such as Enterococcus faecalis, Staphylococcus aureus, Pseudomonas aeruginosa, Bacillus subtilis, Streptococcus species, Actinomyces species and Candida species, among others may remain in root canals [**11**,**12**].

Periodontal and endodontic therapies can be grouped into three broad categories: mechanical debridement and cleaning to eliminate bacteria; treatment aimed at killing or affecting the metabolism of the infectious organism, such as antiseptics and antibiotics, and treatment that affect the environment of the infectious organism [**13**].

Although the antimicrobial efficacy of conventional irrigants is generally recognized, they may produce some complications such as tissue irritation or hypersensitivity [**14**].

Systemic administration has been useful in treating periodontal pockets, but it involves a relatively high dose with repeated intake over a prolonged period of time to achieve the required inhibitory concentrations in the sulcular fluid. This increases the chances of development of resistance, of alteration of commensal flora and of increased potential for adverse effects like allergic/ anaphylactic reaction, gastric disturbances, superinfection, nausea, vomiting, etc. [**15**,**16**].

New approaches involve the use of local drug delivery systems based on microparticles/nanoparticles made from biocompatible polymers. Such devices enable the introduction of antimicrobial agents or other drugs directly in the periodontal pocket, or inside the root canal, and the prolonged release of constant concentrations of these agents for a better control of infections.

Hence, this review aims to evaluate the efficacy of various local drug delivery devices used in intrapocket and intracanal therapy, and summarizes the results of in vitro and in vivo studies demonstrating the major role of these drugs when delivered locally (subgingivally) into the periodontal pockets and in root canals as intracanal medicaments.

**Classification**

**Based on duration of action (Greenstein & Tonetti 2000) [**17**]**

 A) sustained release devices

−Drug delivery for less than 24 hrs

−require multiple applications

−follow first order kinetics

B) controlled delivery devices

−duration of drug release exceeds 24 hrs

−administered once

−follow zero order kinetics

**Characteristics of locally delivered antimicrobials**

Before any antimicrobial agent can be recommended for periodontal or intracanal a number of basic and important conditions have to be fulfilled (Greenstein and Tonetti, 2000) [**17**].

1.Medication must reach intended site of action

2.Remain at adequate concentration

3.Last for a sufficient duration

4.Substantivity.

**Indications**

• Isolated periodontal pockets (>5mm), with successful phase 1 therapy (scaling and root planing),

• Medically compromised patients where surgical therapy is contraindicated or not suggested,

• As an adjunct to mechanical debridement,

• In patients suffering from recurrent or refractory periodontitis,

• As an adjunct to periodontal regenerative procedures,

• Where periodontal surgery is to be avoided and the patient is on supportive periodontal treatment,

• To prevent or control microbial induced inflammation,

• Endodontic failures,

• Patients who have undergone endodontic therapy, but suffer from persistent infections or resistant microorganisms,

• Hypersensitivity to conventional intracanal irrigants like sodium hypochlorite [**14**,**18**,**19**].

**Contraindications**

• Patients with known hypersensitivity reaction to any of the antimicrobials used as local drug,

• Patients susceptible to infective endocarditis who are contraindicated for irrigation devices to avoid the risk of bacteremia,

• Delivery of antimicrobials using ultrasonic devices is contraindicated in asthmatics, infective conditions such as AIDS, tuberculosis and those with cardiac pacemakers [**18**].

**Recent local drug delivery agents and periodontal diseases**

Antimicrobial or antiseptic agents are applied locally for the treatment of periodontitis in combination with mechanical root debridement. Subgingival irrigation with drug solutions provides high concentrations in the periodontal pocket for only short periods. In consequence, repeated oral irrigation is required to exert bactericidal or bacteriostatic effects. The mode by which a drug is delivered to a specific site of action has a significant role on its efficacy. The use of controlled drug release devices may well improve the antimicrobial efficacy in the periodontal pocket, with consequent clinical benefits. The efficacy of drug delivery systems is mainly affected by the biological environment and the properties of the polymer and the drug [**20**]. A multidisciplinary approach to the delivery of therapeutics to target tissues is needed for a precise control of pharmacokinetics and pharmacodynamics of the drug to ensure adequate concentration at the targeted site.

Potential release mechanisms involved for controlled drug release can be: (i) desorption of surface-bound/adsorbed drugs; (ii) diffusion through the carrier matrix; (iii) diffusion (in the case of nanocapsules) through the carrier wall; (iv) carrier matrix erosion; and (v) a combined erosion /diffusion process [**21**]. The mode of delivery primarily controls the drug’s success and failure, as the choice of a drug is often influenced by the way it is administered. Various drug delivery devices for intrapocket therapy include:

1) Fibers- Fibers are thread – like devices placed in periodontal pocket and secured with periodontal dressing, ensuring a sustained release of drug at the site. Fibers used in periodontal disease are of two types – hollow and monolithic fibers. Hollow fibers contain a reservoir of drug, which is released by a simple diffusion through reservoir walls [**22**]. Whereas, to decrease the speed of releasing the drug, the monolithic fibers were developed by impregnating drug into the molten polymers, spinning it on high temperature followed by a quick cooling [**23**]. Aimetti et al [**24**] evaluated the clinical, radiological and microbiological response to the local delivery of tetracycline fibers along with scaling and root planing at sites with persistent periodontal lesions and obtained the greatest advantage in the treatment of up to 12 months following treatment.

2) Films- Films are implantable matrix delivery devices with encapsulation of drug, in a manner that they are distributed throughout the polymer and control release occurs by drug diffusion and/or matrix dissolution or erosion. Films of various polymers have been made for the controlled release of therapeutic agents. The release action depends on the type of polymer used to manufacture the chip. The ease of insertion with minimal pain, control on dosage, dimension and shape of the films make them an ideal device to be used in periodontal pocket. The thickness of the film should not exceed 400 μm as well as it should have sufficient adhesiveness to ensure no interference in maintaining oral hygiene habits. Films that release drugs by diffusion alone are prepared by using water-insoluble non-degradable polymers, whereas those that are released by diffusion and matrix erosion or dissolution use soluble or biodegradable polymers [**25**]. Periochip is the controlled subgingival delivery of chlorhexidine, and was developed by Perio Products Ltd, Jerusalem, Israel. This is an orange brown rectangular chip, rounded at one end. It measures 5 mm x 4 mm x 0.3 mm, weighs about 7.4 mg and contains 2.5 mg of chlorhexidine gluconate, which is incorporated in a biodegradable matrix of hydrolyzed gelatin cross linked with glutaraldehyde. Paolantonio et al [**26**] conducted a study to evaluate clinical and microbiologic effects of chlorhexidine chips when used as an adjunct to scaling and root planing. The adjunctive use of chlorhexidine chip resulted in significant probing depth reduction and a clinical attachment level gain compared to scaling and root planing alone. The results were concomitant with a significant benefit of scaling and root planing plus chlorhexidine treatment on subgingival microflora. In an in vitro study, Perugini et al [**27**] showed that the composite micro material films made of three layers of polymers (chitosan/ PLGA /chitosan), compared to the monolayer films, represent a suitable dosage form to prolong ipriflavone release for 20 days. Jeffcoat et al [**29**] showed a significant change in probing depth and clinical attachment levels after the placement of a biodegradable chlorhexidine gelatin chip in the treatment of adult periodontitis. Natural polymer like gelatin, [**28**] chitosan, [**27**,**29**] collagen [**26**] and synthetic polymers like polyvinyl alcohol (PVA), poly (D,L-lactide-co-glycolide) (PLGA), [**30**] are commonly used to synthesize films.

3) Gels- Gels are injectable semi solid devices containing an adequate concentration of drug and delivered at specific site. They are easy to prepare and administer. They further possess a property of bioadhesivity that enhances the retention time in the periodontal pocket. Salvi et al [**18**] demonstrated a significant reduction in the clinical and microbiological parameters after using elyzol (metronidazole gel) and atridox (doxycycline gel). Perinetti et al [**32**] compared the clinical healing and the microbiological findings following repeated intrasulcular applications of 1% metronidazole or 1% chlorhexidine gels in persistent periodontal pockets previously treated by scaling and root planing and obtained a similar reduction with both gels at 1% concentration.

Novel local drug delivery agents used for the treatment of periodontal diseases are alendronate and simvastatin. Alendronate (4-amino 1-hydroxybutylidine bisphosphonate), a novel bisphosphonate is a very potent inhibitor of bone resorption. The net effect of alendronate on bone formation might be explained by its inhibition of osteoclasts, thus affecting bone maturation and remodeling. Once taken up by bone, alendronate has a prolonged skeletal retention (half-life up to several years) and significant amounts can be released in the resorptive process which may in turn provide protection to the alveolar bone [**33**]. Alendronate gel has been found to increase bone formation on local delivery into the periodontal pocket. In patients with type 2 diabetes mellitus and chronic periodontitis, local delivery of 1% alendronate gel into periodontal pockets resulted in a significant increase in the probing depth reduction, clinical attachment level gain, and improved bone fill compared to placebo gel as an adjunct to scaling and root planning [**34**].

Simvastatin (SMV) is a specific competitive inhibitor of 3-hydroxy-2-methyl-glutaryl coenzyme-A reductase. Pardeep et al [**35**] showed a greater decrease in gingival index and probing depth and a clinical attachment level gain with significant defect fill at sites treated with scaling and root planing plus locally delivered SMV gel in patients with chronic periodontitis.

Bhardawaj et al [**36**] compared and evaluated the clinical effects of topical subgingival application following scaling and root planing of a new biodegradable xanthan based chlorhexidine gel as compared with a gel containing chlorhexidine and metronidazole and scaling and root planing alone. Xanthan based chlorhexidine gel showed the greatest improvements in clinical parameters and a maximum reduction in occurrence of P. gingivalis and an almost similar reduction for Aggregatibacter actinomycetemcomitans and fusobacterium species.

4) Microparticulate system- This system consists of an encapsulation of the drug into a polymer, which dissolves gradually releasing the drug at the target site. It is a highly stable system for the delivery of an optimum concentration of the drug in the pocket. Gopinath et al [**37**] conducted a study to evaluate the effect of a controlled release device containing minocycline microspheres (Arestin TM) on the treatment of chronic periodontitis. The results of this study concluded that treatment with scaling and root planing plus minocycline microspheres (Arestin™) is more effective and safer than scaling and root planing alone in reducing the signs of chronic periodontitis.

5) Nanoparticulate system- Modern drug delivery systems are designed for targeted controlled slow drug release. Up to now, polymer or microparticle-based hydrogels have been applied in dentistry, which can affect the rate of release because of their structure. Recently, intensive research has been performed all over the world to improve the effectiveness of delivery systems. The nanoparticulate system provides several advantages as compared with microspheres, microparticles and emulsion-based delivery systems, including high dispersibility in an aqueous medium, controlled release rate and increased stability. Due to their small size, nanoparticles penetrate regions that may be inaccessible to other delivery systems, such as the deep periodontal pockets. These systems reduce the frequency of administration and further provide a uniform distribution of the active agent over an extended period of time [**22**]. Nanosizing of drugs can lead to a dramatic increase in their absorption and bioavailability leading to a subsequent reduction in drug dose [**38**]. Owing to its small size, it acquires a high dispersibility in an aqueous medium and controlled release rate. The polymer-based nanoparticles were prepared via micellar polymerization, resulting in powder material with particle size in the range of 50–180 nm [**20**]. In an attempt to obtain a novel delivery system adequate for the treatment of periodontal disease, triclosan-loaded polymeric (PLGA, poly-lactic-acid and cellulose acetate phthalate) nanoparticles were prepared by emulsification–diffusion process. A preliminary in vivo study in dogs with induced periodontal defects suggested that triclosan-loaded nanoparticles penetrate through the junctional epithelium [**30**]. These systems reduce the frequency of administration and further provide a uniform distribution of the active agent over an extended period of time [**22**].

**Recent Intracanal local drug delivery systems**

Intracanal local drug delivery agents usually consist of antibiotics to eliminate bacterial infection and non steroidal anti inflammatory drugs to reduce/eliminate post operative pain, after mechanical cleaning has been done i.e. post bio-mechanical preparation (BMP). Many systems of different shapes (sharp cones, long pins or screws) have been proposed for placement in the root canal as a temporary dressing for local drug delivery and healing. Huang et al [**39**] conducted a study in which chlorhexidine-loaded devices were prepared with ethyl cellulose, to form a needle-like device suitable to be inserted in the root canal. Chitosan, PLGA and polymethylmethacrylate have been used as coatings for absorbent paper to examine the controlled release of chlorhexidine digluconate loaded-paper points [**40**].

1) Microparticulate systems- Novel formulations based on microparticles, which appear promising for the accessibility to the conventionally inaccessible parts of the root canal which have been developed. Sousa et al [**41**] developed amoxicillin-loaded microparticles (5–38 μm) by spray-drying. The antimicrobial activity of amoxicillin was preserved when it was encapsulated, and in vitro antimicrobial activity studies demonstrated that the MIC 90 against E. faecalis was achieved over 6 days.

2) Nanoparticulate systems- Use of nanoparticles as local intracanal drug delivery system is also gaining popularity nowadays. Pagonis et al [**42**] used a combination of light and PLGA nanoparticles (150–200 nm) loaded with the photoactive drug methylene blue to treat infections caused by E. faecalis. Chitosan nanoparticles were also found to display a high degree of antibacterial activity against E. faecalis in in-vitro culture, and good compatibility with the endodontic sealer [**43**].

## Conclusions

The management of periodontal diseases and endodontic infections has traditionally focused on the use of anti-infective irrigating solutions and the use of mechanical procedures to eliminate infectious tissue and to hinder disease progression. Several sustained and controlled-release antimicrobials have been developed and tested. Novel drug-loaded microspheres or nanoparticulate systems can be considered as good carriers for the controlled release of several active substances/ antimicrobial agents suited to such specific sites as periodontal pockets or dental cavities. Most have shown positive clinical and microbiological effects, but increased the cost of therapy. The benefits like improved patient compliance and pharmacokinetic response, easy access to the diseased site, and lower drug dosages may overcome the cost factor. Site specific administration of simple injectable formulations of micro/nano particles in the periodontal pocket or in the root canal cavity would reduce the number of treatment sessions for patients, and may serve as an adjuvant to surgical protocols offering a means of saving teeth which is the ultimate goal of any dental treatment. 

**Sources of Funding- None**

**Disclosures- None**
